# The Effect of Walnut Intake on Lipids: A Systematic Review and Meta-Analysis of Randomized Controlled Trials

**DOI:** 10.3390/nu14214460

**Published:** 2022-10-23

**Authors:** Saeed Mastour Alshahrani, Reham M. Mashat, Diaa Almutairi, Alaa Mathkour, Sahar Saad Alqahtani, Amirah Alasmari, Abdullah Hassan Alzahrani, Reem Ayed, Mohammed Yahya Asiri, Alsanussi Elsherif, Abdullah Alsabaani

**Affiliations:** 1 Department of Public Health, College of Applied Medical Sciences, King Khalid University, Khamis Mushait 62529, Saudi Arabia; 2 Nutrition and Food Sciences, College of Home Economics, King Khalid University, Abha 62529, Saudi Arabia; 3 Prince Sultan Military College of Health Sciences, Dhahran 34247, Saudi Arabia; 4 Ministry of Health, Riyadh 12613, Saudi Arabia; 5 Public Health Authority, Riyadh 13351, Saudi Arabia; 6 Department of Family and Community Medicine, Faculty of Medicine, University of Benghazi, Benghazi 1308, Libya; 7 Department of Family and Community Medicine, College of Medicine, King Khalid University, Abha 62529, Saudi Arabia

**Keywords:** walnuts, dyslipidemia, total cholesterol (TC), triglyceride (TG), low density lipoprotein cholesterol (LDL-C), randomized controlled trials, meta-analysis

## Abstract

Cardiovascular diseases (CVD) are the leading causes of death worldwide. Dyslipidemia is a cardiometabolic risk factor of CVD, yet it can be modifiable. Walnuts have been suggested as a dietary intervention to improve the lipid profile. Therefore, we reviewed the literature to assess the evidence linking walnut intake to the improvement of blood lipids, including total cholesterol (TC), low-density lipoprotein (LDL-C) cholesterol, high-density lipoprotein (HDL-C) cholesterol, and triglycerides (TG). PubMed and Embase databases were searched from 2010 up to March 2022. We limited our search to randomized controlled trials conducted on humans and published in English during the specified period. Cochrane’s risk of bias tool for interventional studies was used. A random-effects model was used for the meta-analysis, and weighted mean differences were obtained (WMD) Thirteen trials from the U.S., Europe, and Asia were included. Walnut intake was associated with significant reductions in TC (WMD: −8.58 mg/dL), LDL-C (WMD: −5.68 mg/dL), and TG (WMD: −10.94 mg/dL). Walnut consumption was not associated with HDL-C. Subgroup analysis showed that overweight/obese and those with comorbidities had more lipid improvement. A longer trial duration did result in further improvements. However, our results may be prone to bias due to extraneous confounding factors. Additionally, levels of heterogeneity were considerable for some outcomes of interest. Results from this meta-analysis provide evidence for the health benefits of walnuts on blood lipids. Walnuts possibly reduce the risk of CVD; thus, they can be successfully added to a dietary pattern to enhance health benefits.

## 1. Introduction

According to the World Health Organization (WHO), cardiovascular diseases (CVD)—specifically, ischemic heart diseases (IHD) and stroke—have been identified as the leading cause of death worldwide [1]. The cardiometabolic risk factors for CVD include metabolic syndrome, elevated blood pressure, obesity, diabetes mellitus, and dyslipidemia [2]. However, these factors can be modified in most situations to reduce the CVD risk [2,3]. One important modifiable risk factor of CVD is dyslipidemia.

Dyslipidemia is defined as when an individual has increased plasma levels of either total cholesterol (TC), low-density lipoprotein cholesterol (LDL-C), or triglycerides (TG), or has decreased plasma levels of high-density lipoprotein cholesterol (HDL-C) [4]. Several clinical studies have been conducted to assess the effectiveness of lipid-lowering medications in reducing CVD-related events [5]. On the other hand, other studies have adopted non-drug approaches, including physical activity programs and dietary modification, to improve the lipid profile and reduce CVD risk [6,7,8,9].

Several dietary factors may contribute to dyslipidemia, such as high intakes of carbohydrates and fat [10,11,12]. Regarding the fat, however, the type of fat consumed plays an essential role in the development of dyslipidemia. A recent study found that the intakes of trans-fatty acids (TFA) and saturated fatty acids (SFA) had been positively associated with dyslipidemia [13]. On the other hand, diets that are high in monounsaturated fatty acids (MUFA) and polyunsaturated fatty acids (PUFA), such as avocado and canola oil, had beneficial effects on reducing the risk of dyslipidemia [14,15]. Therefore, many researchers have become interested in dietary modifications as an interventional approach to treat dyslipidemia.

The plant-based diet has been associated with a positive impact on dyslipidemia [16,17]. The anti-inflammatory micronutrients, particularly MUFA and PUFA, may explain such a positive impact of the plant-based diet [18]. Nuts are an example of MUFA and PUFA-enriched plant-based diet [19]. Nuts also are rich in several bioactive nutrients such as dietary fiber, vitamins, minerals, and phytosterols [20]. In addition, nuts have been associated with beneficial effects on CVD risk factors [21]. A meta-analysis of 61 controlled intervention trials found considerable reductions in TC, LDL-C, and TG attributed to nut consumption [22].

One of the most popular health-promoting nuts is walnuts, which not only are rich in PUFA, but also contain the highest omega-3:omega-6 ratio—the more desirable ratio for CVD risk reduction—amongst all types of nuts [23,24]. Despite the various forms and cultivars of walnuts, all have shown distinct antioxidant characteristics [25]. As a result, walnuts have been associated with a reduced risk of CVD-related events and their risk factors [26,27,28]. Furthermore, regarding its association with dyslipidemia, walnut intake was significantly associated with reductions in TC and LDL-C by approximately 10 and 9 mg/dL, respectively [28].

Despite the potential health benefits of walnut intake, one may argue that consistent consumption of walnuts may lead to unfavorable outcomes such as weight gain or obesity, a risk factor for CVD. Therefore, it is of interest to review the risk–benefit of walnut consumption as a part of daily dietary habits aiming to reduce the risk of CVD and its risk factors, especially dyslipidemia. The effects of walnuts in improving the lipid profile have been previously reviewed [29]. Although only controlled trials were included in that study, the review has included studies since 1993. Ever since, the methodologies of clinical trials have been updated significantly over the decades, primarily due to the technologies utilized in clinical research for compliance assurance and subjects’ contact.

In this review, we focused on the recently published randomized controlled trials that have explored the effect of walnut intake (as an intervention diet) in comparison with a control diet (a non-walnut-included diet) on the lipid profile, including total cholesterol (TC), low-density lipoprotein cholesterol (LDL-C), high-density lipoprotein cholesterol (HDL-C), and triglycerides (TG). Results from the current study should provide additional insights on the risk–benefit assessment of recommending walnut consumption as a part of the habitual diet to improve the lipid profile and reduce CVD risk.

## 2. Materials and Methods

In this systematic review and meta-analysis, we followed the Cochrane Handbook for Systematic Reviews of Interventions guidelines and Preferred Reporting Items for Systematic Reviews and Meta-analyses (PRISMA) [30,31]. We limited our search to randomized controlled trials conducted on humans and published in English from 2010 to March 2022. No systematic reviews or meta-analyses were included, nor did we include non-randomized trials. We believe that the methodology of controlled trials has become more sophisticated over the years, particularly in the last decade, due to the remarkable evolution of technology. Modern technology, including mobile phone use, has been used in interventional studies for compliance assurance and subject communications.

### 2.1. Search Strategy

A systematic literature search was conducted in PubMed and Embase for randomized controlled trials investigating the effect of walnut intake (as an intervention diet) compared with a control diet (a non-walnut-included diet) on the lipid profile. We used the following search terms to search the targeted databases: (*(Juglans* OR *walnuts)* AND *(triglyceride* OR *cholesterol* OR *lipoprotein* OR *low-density lipoprotein* OR *LDL OR LDL-C* OR *high-density lipoprotein* OR *HDL OR HDL-C OR lipid profile)* AND *(randomized OR randomised)* NOT *(systematic review OR meta-analysis)*).

### 2.2. Study Selection

In order to be included in this review, a trial must have assessed the effect of walnut consumption—as a part of a daily diet—compared with a control diet (a diet without walnuts) on lipids. In addition, lipids must be the primary outcomes of interest of the trial to be included. Studies that included walnut intake along with other mixed nuts were excluded as it would be nearly impossible to extract the sole effect of walnut apart from other nuts. Studies must have also reported baseline and follow-up values for the outcomes of interest. That is, the mean change from baseline for intervention and control groups or the mean difference between intervention and control groups for at least one lipid variable (e.g., TC, LDL-C, HDL-C, TG) must have been reported. Furthermore, studies must have specifically tested walnut-based interventions and clearly stated the amount and frequency of walnuts given or instructed to be consumed in the diet.

### 2.3. Data Extraction

Studies that met inclusion criteria were thoroughly reviewed and extracted independently. We extracted the following information from the included studies: First author’s last name, year of publication, location of the study, sample size, study design, participants’ characteristics (e.g., mean age, gender distribution, mean body mass index, when available, health status), duration of treatment, intervention type and dose, controls details, and the results of the effects.

### 2.4. Quality Assessment

We used Cochrane’s risk of bias tool for interventional studies [32]. The tool aims to assess seven components of each trial as follows: (1) Random sequence generation, (2) allocation concealment, (3) blinding of participants and personnel, (4) blinding of outcome assessors, (5) incomplete outcome data, (6) selective reporting, and (7) other biases. We independently assessed the quality of the included studies in terms of the methodological approaches applied in each study. The risk in each component was categorized into low risk, unclear risk, or high risk. The final decision was made after the investigators unanimously agreed.

### 2.5. Statistical Analysis

We used Cochrane RevMan 5.4 software (Review Manager [RevMan; computer program] Version 5.4. The Cochrane Collaboration, 2020) for this current meta-analysis. We specifically used the generic inverse variance weighting method with a random-effects model to account for potential variabilities between studies; however, we additionally explored the fixed-effects model for comparison. We obtained the effect’s weighted mean difference (WMD) and 95% confidence intervals (CI). We calculated the mean change from baseline to follow-up for intervention and control groups by subtracting the mean at the baseline from the mean at the end of the follow-up. We used the control group with a diet comparable to the intervention group—but with no nut’s components—if there was more than one comparison group. If only the mean difference were reported, we used it for the intervention group and set the mean of the controls as zero. If not reported, standard deviation (SD) was obtained from standard error (SE) or CI. Lipid values reported in millimole per liter (mmol/L) were converted into milligrams per deciliter (mg/dL) by multiplying TC, LDL-C, and HDL-C by 38.67 and multiplying TG by 88.57. Heterogeneity was assessed using Cochrane’s Q test and *I*^2^. According to Cochrane Handbook for Systematic Reviews of Interventions [30], heterogeneity might not be substantial when *I*^2^ is 0% to 40%, heterogeneity is moderate when *I*^2^ is 30% to 60%, heterogeneity is substantial when *I*^2^ is 50% to 90%, and heterogeneity is considerable when *I*^2^ is 75% to 100%. We also conducted a subgroup analysis to explore the change in the walnut consumption effect on lipids according to the several characteristics of the participants in the trials. Subgroups of interest were health status (healthy vs. those with comorbidities), mean age in years (≥55 vs. <55), mean body mass index (BMI) in kg/m^2^ (>25 vs. ≤25), gender distribution (majority females vs. majority males or almost equal gender distribution), baseline lipid levels in mg/dL (TC: ≥200 vs. <200; LDL-C: ≥130 vs. <130; HDL-C: ≥50 vs. <50; TG: ≥150 vs. <150), and trial duration (>12 weeks vs. 12 weeks or less). In order to further investigate the potential source of heterogeneity, sensitivity analysis was conducted by removing each study once at a time and then recalculating the effect and assessing the magnitude of the change. Publication bias was evaluated through Begg’s funnel plots by checking any skewness (asymmetry) on either side of the plots [33].

## 3. Results

### 3.1. Study Selection

**Figure 1:**
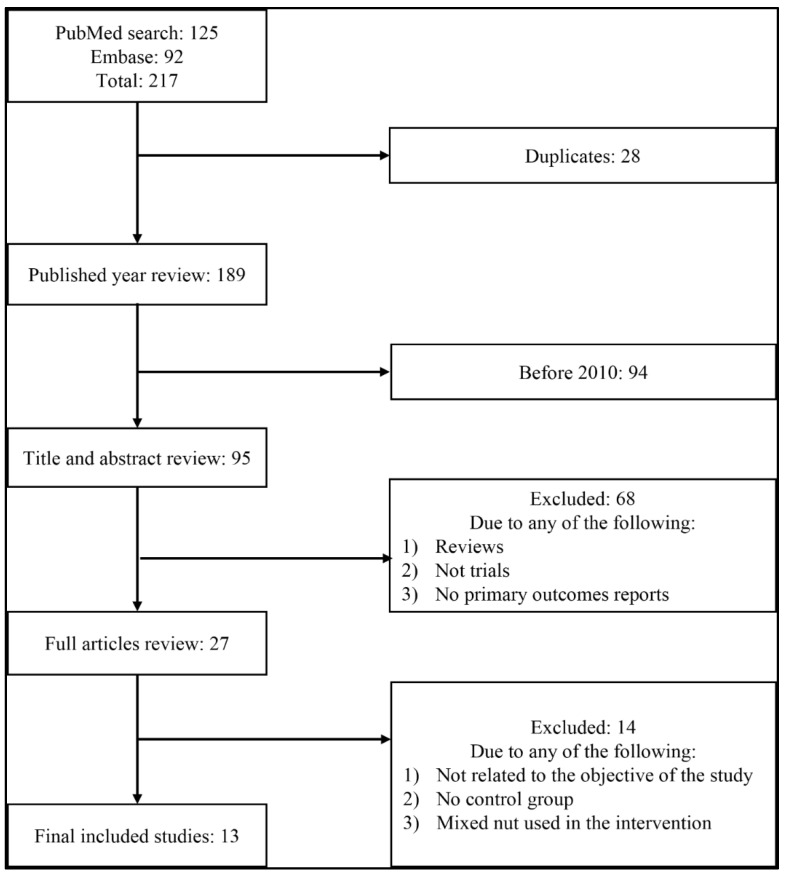
PRISMA flowchart of study selection process for the meta-analysis.

Studies were selected after a comprehensive search of the literature in PubMed and Embase. The flowchart presented in Figure 1 demonstrates the sequence of the study selection approach. Initially, our search terms retrieved 217 articles from both search engines (125 from PubMed and 92 from Embase); we excluded 28 duplicate articles. We further excluded 94 articles as they were published before 2010. An additional 68 articles were excluded after titles and abstracts review as they were either review articles, not trials, or primary outcomes not reported. Furthermore, we excluded 14 articles after the full-article review process as they either were not strongly related to the study’s objective, did not have a control group that matches our study inclusion criteria, or used mixed nuts besides walnut in the intervention. The final included trials in this review were 13 randomized controlled trials [34,35,36,37,38,39,40,41,42,43,44,45,46] (Figure 1).

### 3.2. Characteristics of the Included Studies

Thirteen trials were included in this meta-analysis (Table 1). Five of the 13 included trials were conducted in the U.S. [35,36,42,43,45]; three from Germany [38,39,41]; two from Iran [37,40]; and one from UK [34], Spain [46], and China [44]. Ten trials were cross-over designs [34,35,36,38,39,41,42,43,45,46]; and three were parallel designs [37,40,44]. The trials durations for most included trials ranged from 6 to 16 weeks [34,35,36,37,38,39,40,41,44,45,46]; however, one trial lasted for 52 weeks [43]. Walnut dosage used for the intervention ranged from 15–99 g/d in most trials [34,35,36,38,39,40,41,42,43,44,45,46], except for one trial that used 100 mg Juglans regia leaves extract powder capsule twice a day [37]. One trial was conducted only on males [34], while the others were conducted on males and females [35,36,37,38,39,40,41,42,43,44,45,46]. Five trials were conducted on healthy participants [34,36,38,41,43]; three on diabetic patients [37,40,45]; two on overweight/obese patients [35,39]; one on hypercholesteremic patients [46]; one on those with metabolic syndrome [44]; and one on those with a cardiovascular disease [42]. The average age of the participants in all trials ranged from 23–63 years; the average BMI ranged from 24–33 kg/m^2^; sample sizes of all trials ranged from 30–212 participants.

### 3.3. Quality Assessment

**Figure 2:**
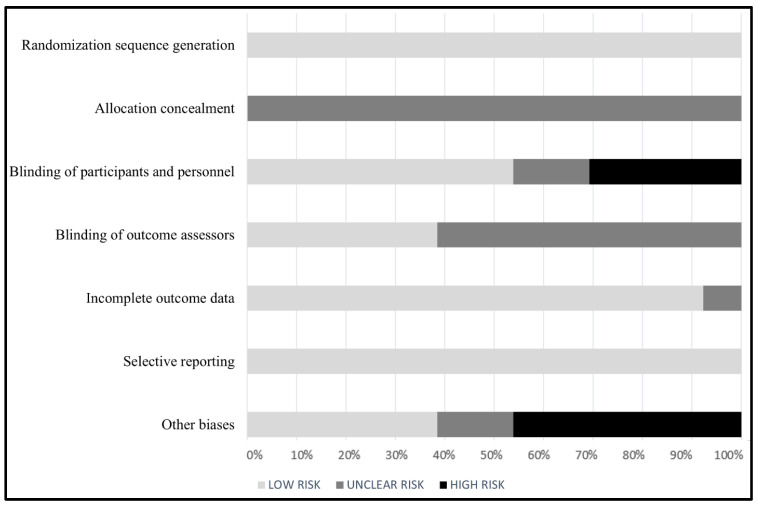
Quality assessment of included studies according to Cochrane risk of bias tool.

Figure 2 illustrates the results of the quality assessment of included studies. Almost all studies demonstrated a low risk of bias in random sequence generation, incomplete outcomes results, and selective reporting [34,35,36,37,38,39,40,41,42,43,44,45,46]. Regarding allocation concealment, all studies had an unclear risk [34,35,36,37,38,39,40,41,42,43,44,45,46]. For blinding criteria, approximately one-quarter of the studies included had a high risk of bias regarding the blinding of participants and personnel [38,39,42,44], while all included studies had either an unclear risk or low risk of bias regarding the blinding of outcome assessors [34,35,36,37,38,39,40,41,42,43,44,45,46]. Finally, several studies had a high risk of other biases as they reported potential sources of biases related to compliance to the intervention, generalizability, or possible measurement error [35,36,40,41,42,45].

### 3.4. Meta-Analysis Results

**Figure 3:**
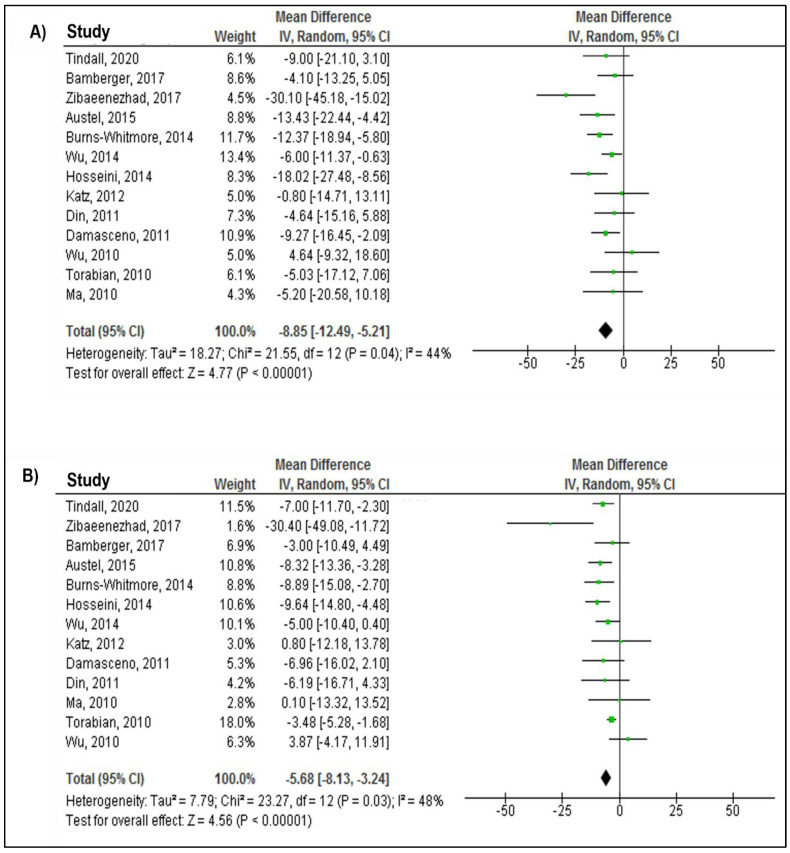

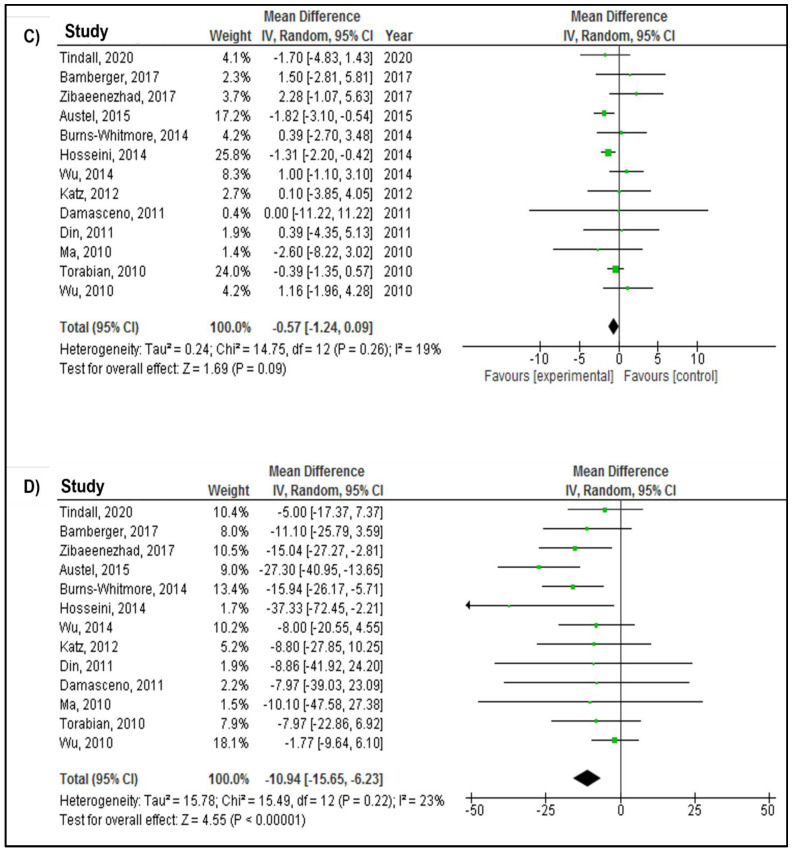
Forest plot presenting mean difference (MD) and 95% confidence intervals for the effect of walnut consumption on (**A**) TC (mg/dL), (**B**) LDL−C (mg/dL), (**C**) HDL−C (mg/dL), and (**D**) TG (mg/dL) [34,35,36,37,38,39,40,41,42,43,44,45,46].

Figure 3 presents the results of walnut intake effects on TC, LDL-C, HDL-C, and TG from the random-effects model. The effect of walnut intake on all four lipid outcomes was evaluated in all included studies (515 in the intervention group and 522 in the control group). The overall effect of the weighted mean difference (WMD) showed significant reductions in TC (WMD: −8.58 mg/dL; 95% CI: −12.94, −5.21; *p* < 0.0001), LDL-C (WMD: −5.68 mg/dL; 95% CI: −8.13, −3.24; *p* < 0.0001), and TG (WMD: −10.94 mg/dL; 95% CI: −15.65, −6.23; *p* < 0.0001). Walnut consumption was not associated with HDL-C (WMD: −0.57 mg/dL; 95% CI: −1.24, 0.09; *p* = 0.09) (Figure 3). The heterogeneity tests revealed significant between-study heterogeneity for the association of walnut intake with TC (P-heterogeneity = 0.04; *I*^2^ = 44%) and LDL-C (P-heterogeneity = 0.03; *I*^2^ = 48%), while non-significant between-study heterogeneity was observed for the association of walnut intake with HDL-C (P-heterogeneity = 0.26; *I*^2^ = 19%) and TG (P-heterogeneity = 0.22; *I*^2^ = 23%) (Figure 3).

### 3.5. Subgroup Analysis

Results from subgroup analysis have provided additional insights into the effect of walnut consumption on lipids (Table 2). Regarding TC, a lowering effect of walnuts was observed among those with comorbidities compared to healthy participants (−10.45 vs. −7.24). Further, a much-lowering effect was observed among those with a mean BMI > 25 kg/m^2^ compared to those with 25 kg/m^2^ or less (−12.98 vs. −6.50). Those with baseline TC of <200 mg/dL showed a much-lowering effect compared to those with ≥200 mg/dL (−11.17 vs. −7.74). As for trial duration, trials of 12 weeks or less showed a much-lowering effect compared to longer than 12 weeks trials (−10.88 vs. −7.26). Regarding LDL-C, a much-lowering effect was observed among those with mean BMI > 25 kg/m^2^ compared to those with 25 kg/m^2^ or less (−8.28 vs. −3.76). Additionally, those with a baseline LDL-C of <130 mg/dL showed a much-lowering effect compared to those with ≥130 mg/dL (−7.43 vs. −4.94). Trials of 12 weeks or less showed a lowering effect compared to longer than 12 weeks trials (−6.69 vs. −4.21). Regarding HDL-C, lowering effects of walnut on HDL-C were observed among those with comorbidities and overweight. As for the effect of walnuts on TG, a much-lowering effect was observed among those with mean BMI > 25 kg/m^2^ compared to those with 25 kg/m^2^ or less (−15.12 vs. −7.81). Interestingly, trials with mostly females showed a much-lowering effect compared to trials with mostly males or almost equal gender distribution (−14.72 vs. 6.96). Finally, trials of 12 weeks or less showed a much-lowering effect compared to longer than 12 weeks trials (−14.17 vs. −10.12) (Table 2).

### 3.6. Sensitivity Analysis

We conducted sensitivity analyses to assess every study’s effect on the overall effect size. That is, each study was removed from the analysis and reassessed the change in the effect. There was no substantial impact of any study on the overall effect of walnuts on TC, LDL-C, HDL-C, and TG. In addition to using a random-effects model, we reran the meta-analysis using a fixed-effects model; no considerable change was observed between the two models.

### 3.7. Publication Bias

Through a visual inspection of Begg’s funnel plot, we believe that there was no evidence of publication bias for studies investigating the effect of walnut consumption on blood lipids. The plots did not illustrate any severe skewness or asymmetry around the effect sizes of the outcomes of interest (Supplementary Materials Figure S1).

## 4. Discussion

This systematic review and meta-analysis explored the effect of walnut intake on the lipid profile (e.g., TC, LDL-C, HDL-C, and TG) in 13 randomized controlled trials. We found that the intake of walnuts was significantly associated with improved TC, LDL-C, and TG levels. However, there was no significant impact of walnut consumption on HDL-C. Subgroup analysis indicated that those who had BMI > 25 kg/m^2^, comorbidities, and normal levels of TC and LDL-C had more improvements in TC and LDL-C levels post-intervention as compared to those who had BMI ≤ 25 kg/m^2^, no comorbidities, and abnormal levels of TC and LDL-C, respectively. Additionally, those with BMI > 25 kg/m^2^ had greater improvements in TG levels post-intervention than those with BMI ≤ 25 kg/m^2^. Furthermore, we found that the studies with a majority of female participants exhibited a much-lowering effect of TG compared to studies with a majority of male participants or equal gender distribution. Finally, trials that lasted more than 12 weeks did not appear to result in more improvement effects on lipids. In fact, trials conducted within 12 weeks or less had much more lowering effects on TC, LDL-C, and TG levels than trials that lasted more than 12 weeks.

Our results are comparable to the latest meta-analysis results conducted on 26 studies investigating the effect of walnut intake on lipids, especially the walnut intake effect on TC and LDL-C [29]. That is, Guasch-Ferré et al. reported approximately 7 and 5.5 mg/dL significant reductions in TC and LDL-C levels post-intervention as compared to our results where we found reductions of 8.85 and 5.68 mg/dL, respectively [29]. Our findings for TC were also consistent with an earlier meta-analysis, in which Banel et al. found approximately 10 mg/dL reduction compared to ours (8.85 mg/dL) [28]. However, they found a much-lowering effect for LDL-C compared to our results, which could be attributed to a potential bias resulting from non-randomization as indicated in one of the trials they included [47]. Regarding TG, however, a considerable difference in the findings between ours and the results of Guasch-Ferré et al. was noticed. That is, we found a 10.94 mg/dL reduction post-intervention compared to a 4.69 mg/dL reduction reported by Guasch-Ferré et al. This substantial difference between the two findings could be attributed to the potential bias of the trials included in Guasch-Ferré et al. study. In fact, two of the trials they included in their review (from before 2010) had reported concerns regarding randomization and compliance [47,48].

Regarding the subgroup analysis, our findings were not entirely consistent with Guasch-Ferré et al. As for trial duration, both meta-analyses found a much-lowering effect of walnut intake on TC and LDL-C for the trials that were conducted for short durations. This may raise queries regarding compliance challenges during the long duration trials. Regarding the subgroup analysis by BMI, however, Guasch-Ferré et al. found that those with normal BMI had more improvements in TC and LDL-C compared to overweight or obese individuals. In contrast, we found much-lowering effects on TC, LDL-C, and TG among overweight or obese individuals compared to those with normal weight. Such a discrepancy could be related to the trials included in Guasch-Ferré et al. that were conducted prior 2010, in which many were conducted among obese individuals [48,49,50,51]. Furthermore, the more improvement effects among overweight or obese as compared to normal weight in our study were also observed in a trial that found an immediate improvement in the lipid profile among obese participants [52]. Interestingly, we also found that those with comorbidities showed much-lowering effects of walnuts on TC and LDL-C as compared to healthy participants. This could be due to the dosage variations between studies composed of healthy participants and studies composed of individuals with comorbidities. That is, the walnut dosage used in trials with healthy participants ranged between 15 and 64 g/d, while it ranged between 30 and 99 g/d in trials composed of individuals with comorbidities. Higher dosage of walnut among participants with comorbidities may have resulted in a much-lowering effect as compared to healthy participants.

Several potential mechanisms could explain the positive effect of walnut consumption on the lipid profile. Walnuts are rich in multiple beneficial nutrients, including polyunsaturated fatty acids (PUFA) (e.g., linoleic and α-linoleic acids), dietary fiber, proteins, antioxidants, vitamins, phytosterol, and minerals such as potassium, calcium, and magnesium [53]. Polyunsaturated fatty acids (PUFA), particularly α-linoleic acids, play an essential role in the uptake of LDL particles, as increased α-linoleic acids enhances LDL receptors to activate, leading to speedy removal of LDL-C from the plasma [54]. A similar effect of walnuts has also been observed for other nuts such as hazelnuts, almonds, and pistachios [55,56,57]. Another potential mechanism could be attributed to the fiber components that existed in walnuts, as they may have contributed to increased fecal bulk, reduced transit time in the intestines, and reduced calorie consumption [58]. Additionally, walnut intake may help in weight management as it has been associated with increased satiation, which might be a reason for lowering blood lipid levels [59]. Finally, walnuts positively impact the gut microbiota, as walnuts may perhaps upregulate the functioning of the probiotics, besides their conversion of bile acid to secondary bile acids, which may have contributed to LDL-C reduction [60,61].

This review evaluates the most recent and comprehensive published reports to estimate the role of walnut intake on the blood lipid profile. We only focused on the latest randomized controlled trials because they aimed to minimize several sources of bias (e.g., selection and confounding biases) by utilizing more advanced tools and methodological approaches [62]. Additionally, we believe that the recent literature, especially in randomized clinical trials, would result in less variations between studies yielding an inconsiderable amount of heterogeneity. Further, the era of mobile phone use in the last decade has contributed to a remarkable evolution in lifestyle interventional trials, including dietary interventions [63]. Such evolution would likely increase the participants’ compliance and protocol commitment.

Nonetheless, the results from this study may be prone to some sources of bias, including extraneous confounding factors, despite our design approach that limits only randomized trials to be included. The widely varied dosage range of walnuts used in the trials included (15–99 g/d) may have contributed to an increased amount of error in this analysis. In fact, levels of heterogeneity were considerable for some outcomes of interest, which could be attributed to the wide dosage range and geographical distribution of the trials included. However, the sensitivity analysis results yield relatively similar findings to the overall results. Furthermore, our results may lack generalizability due to the different dietary backgrounds of the trial participants. For example, the effect of walnuts on lipids among those with a Western diet background may undertake a different pathway among those with Asian or Mediterranean diet backgrounds. Additionally, these results may not be generalizable to those with other diseases than those reported in the included trials. In some circumstances, walnuts could be harmful to those with severe allergic reactions [64].

### Public Health Implications

Dyslipidemia is a cardiometabolic risk factor for CVD, yet it can be modified in most cases [2]. Walnut consumption may be a great contributor to treating dyslipidemia. Not only can walnuts improve the lipid profile, but they also positively impact other metabolic risk factors such as metabolic syndrome, hypertension, and blood glucose [65,66,67]. In addition, walnut intake was positively associated with improved cognitive functions and longevity [68,69]. Furthermore, walnuts are safe—in most cases—and they can be incorporated into the habitual daily diet [70]. Regarding the concerns with weight gain due to walnut consumption, there is no conclusive evidence indicating that they can increase body weight [71]. Therefore, walnuts may be recommended to individuals at high risk of CVD development in clinical settings in order to modify their risk positively.

## 5. Conclusions

The current meta-analysis of randomized controlled trials provides additional evidence for the health benefits of walnut intake on the levels of blood lipids and supports the findings of epidemiologic trials. Despite the fact that walnuts are an extremely energy-dense food, walnut intake possibly reduces the risk of CVD; thus, they can be successfully added to a dietary pattern as a health-promoting diet.

## Figures and Tables

**Table 1:** Characteristics of included studies in the meta-analysis.

Study	Country	Study Design	Sample Size (n)	Participants	Mean Age (yrs)	Mean BMI (kg/m^2^)	Gender (M%, F %)	Mean Baseline Lipids (mg/dL)	Trial Duration (wks)	Intervention		
										Treatment	Dose	Control
Tindall, 2020 [42]	USA	Crossover	34	Cardiovascular disease patients	44	30	62, 38	TC: 182 LDL-C: 115 HDL-C: 45 TG: 111	18	Walnut diet	57–99 g/d	walnut fatty acid–matched diet that did not contain walnut
Bamberger, 2017 [41]	Germany	Crossover	194	Healthy	63	25	31, 69	TC: 232 LDL-C: 146 HDL-C: 69 TG: 101	16	Walnut-enriched diet (shelled walnut)	43 g/d	Nut-free control diet
Zibaeenezhad, 2017 [40]	Iran	Parallel	100	Type II diabetic patients	56	27	59, 41	TC: 234 LDL-C: 144 HDL-C: 48 TG: 194	12	4 walnut oil capsules containing Persian walnut (Juglans Regia L.) oil	15 cc/d	4 placebo capsules containing distilled water
Austel, 2015 [39]	Germany	Crossover	212	Overweight/Obese	52	30	18, 82	TC: 206 LDL-C: 134 HDL-C: 62 TG: 125	12	Walnuts and walnut oil	Two portions	A modified Mediterranean-type diet
Wu, 2014 [38]	Germany	Crossover	40	Healthy	60	25	25, 75	TC: 222 LDL-C: 135 HDL-C: 72 TG: 89	16	walnut-enriched diet (shelled walnuts)	43 g/d	Western diet
Hosseini, 2014 [37]	Iran	Parallel	61	Type II diabetic patients	55	27	46, 54	TC: 192 LDL-C: 105 HDL-C: 40 TG: 162	12	Persian Juglans regia leaves extract powder capsule before meal	100 mg twice a day	Placebo capsule
Burns-Whitmore, 2014 [36]	USA	Crossover	20	Healthy	38	23	20, 80	TC: 185 LDL-C: 109 HDL-C: 49 TG: 100	16	Walnuts eaten raw or used on salads, muffins, etc.	28 g/d	Standard egg diet
Katz, 2012 [35]	USA	Crossover	46	Overweight/Obese	57	33	39, 61	TC: 205 LDL-C: 121 HDL-C: 53 TG: 157	16	Walnut-enriched ad libitum diet (shelled, unroasted English walnuts)	56 g/d	Ad libitum diet without walnuts
Din, 2011 [34]	UK	Crossover	30	Healthy	23	25	Males	TC: 178 LDL-C: 104 HDL-C: 51 TG: 108	8	Walnut supplements	15 g/d	No walnuts
Damasceno, 2011 [46]	Spain	Crossover	18	Hypercholesteremic	56	26	50, 50	TC: 272 LDL-C: 196 HDL-C: 63 TG: 120	12	Spanish grown walnuts (Serr/Chandler variety)	40–65 g/d	Virgin olive oil-based diet
Wu, 2010 [44]	China	Parallel	189	Metabolic syndrome patients	48	25	56, 44	TC: 224 LDL-C: 166 HDL-C: 50 TG: 176	12	Walnuts supplementation	30 g/d	Healthy lifestyle counseling diet
Torabian, 2010 [43]	USA	Crossover	87	Healthy	54	27	44, 56	TC: 220 LDL-C: 131 HDL-C: 59 TG: 123	52	Walnut-supplemented diet	28–64 g/d	Habitual (control) diet
Ma, 2010 [45]	USA	Crossover	24	Type II diabetic patients	58	33	42, 58	TC: 183 LDL-C: 103 HDL-C: 56 TG: 124	8	Walnut-enriched ad libitum (shelled, unroasted English walnuts)	56 g/d	Ad libitum diet without walnuts

**Table 2:** Subgroup analyses of walnut consumption on the lipid profile.

		WMD (95% CI)	*p* for Effect	*p* for Heterogeneity	*I* ^2^
**Subgroups for TC**					
Overall effect		−8.85 (−12.49, −5.21)	<0.00001	0.04	44%
Health status	Healthy	−7.24 (−10.65, −3.82)	<0.0001	0.50	0%
	With comorbidities	−10.45 (−16.58, −4.32)	0.0008	0.02	57%
Mean age	≥55	−9.80 (−15.57, −4.04)	0.0009	0.02	60%
	<55	−8.32 (−13.09, −3.55)	0.0006	0.25	25%
Mean BMI	>25	−12.98 (−19.19, −6.77)	<0.0001	0.06	52%
	≤25	−6.50 (−9.75, −3.25)	<0.0001	0.42	0%
Gender distribution	Majority females	−8.03 (−11.58, −4.48)	<0.00001	0.36	9%
	Majority males or almost equal gender distribution	−10.01 (−16.70, −3.31)	0.003	0.02	61%
Baseline TC	≥200	−7.74 (−12.84, −2.63)	0.003	0.03	54%
	<200	−11.17 (−15.71, −6.64)	<0.00001	0.36	7%
Trial duration	>12 weeks	−7.26 (−10.62, −3.90)	<0.0001	0.55	0%
	12 weeks or less	−10.88 (−17, 48, −4.28)	0.001	0.02	61%
**Subgroups for LDL-C**					
Overall effect		−5.68 (−8.13, −3.24)	<0.00001	0.03	48%
Health status	Healthy	−4.01 (−5.60, −2.42)	<0.00001	0.54	0%
	With comorbidities	−6.19 (−10.49, −1.90)	0.005	0.02	58%
Mean age	≥55	−6.32 (−10.75, −1.90)	0.005	0.09	45%
	<55	−5.24 (−8.28, −2.20)	0.0007	0.06	53%
Mean BMI	>25	−8.28 (−11.94, −4.63)	<0.00001	0.16	37%
	≤25	−3.76 (−5.91, −1.60)	0.0006	0.33	14%
Gender distribution	Majority females	−6.04 (−8.82, −3.26)	<0.0001	0.51	0%
	Majority males or almost equal gender distribution	−6.04 (−10.03, −2.05)	0.003	0.006	67%
Baseline LDL-C	≥130	−4.94 (−8.52, −1.35)	0.007	0.02	60%
	<130	−7.43 (−10.21, −4.65)	<0.00001	0.58	0%
Trial duration	>12 weeks	−4.21 (−5.72, −2.70)	<0.00001	0.42	0%
	12 weeks or less	−6.69 (−11.83, −1.54)	0.01	0.02	61%
**Subgroups for HDL-C**					
Overall effect		−0.57 (−1.24, 0.09)	0.09	0.26	19%
Health status	Healthy	−0.04 (-.85, 0.77)	0.92	0.73	0%
	With comorbidities	−1.07 (-.1.91, −0.23)	0.01	0.32	14%
Mean age	≥55	−0.02 (−1.39, −1.34)	0.97	0.17	33%
	<55	−0.74 (−1.57, 0.09)	0.08	0.33	13%
Mean BMI	>25	−1.22 (−2.04, −0.40)	0.004	0.34	11%
	≤25	−0.01 (−0.79, 0.76)	0.97	0.76	0%
Gender distribution	Majority females	−0.37 (−1.77, 1.02)	0.60	0.19	33%
	Majority males or almost equal gender distribution	−0.59 (−1.39, 0.21)	0.15	0.30	17%
Baseline HDL-C	>50	−0.58 (−1.36, 0.21)	0.15	0.37	8%
	≤50	−0.26 (−1.70, 1.19)	0.73	0.14	42%
Trial duration	>12 weeks	−0.15 (−0.93, 0.63)	0.71	0.70	0%
	12 weeks or less	−0.91 (−1.39, 0.11)	0.08	0.24	25%
**Subgroups for TG**					
Overall effect		−10.94 (−15.65, −6.23)	<0.00001	0.22	23%
Health status	Healthy	−11.50 (−17.71, −5.29)	0.0003	0.87	0%
	With comorbidities	−11.92 (−20.11, −3.73)	0.004	0.05	50%
Mean age	≥55	−11.87 (18.46, −5.27)	0.0004	0.83	0%
	<55	−10.87 (−19.14, −2.61)	0.010	0.03	59%
Mean BMI	>25	−15.12 (−23.91, −6.33)	0.0007	0.17	36%
	≤25	−7.81 (−12.64, −2.97)	0.002	0.56	0%
Gender distribution	Majority females	−14.72 (−20.62, −8.81)	<0.00001	0.41	2%
	Majority males or almost equal gender distribution	−6.96 (−12.50, −1.42)	0.01	0.39	5%
Baseline TG	≥150	−10.17 (−20.98, 0.64)	0.07	0.10	52%
	<150	−12.45 (−17.48, −7.43)	<0.00001	0.49	0%
Trial duration	>12 weeks	−10.12 (−15.51, −4.72)	0.0002	0.83	0%
	12 weeks or less	−14.17 (−24.74, −3.60)	0.009	0.04	55%

## Data Availability

All reported data are available in the manuscript.

## Supplementary Materials

The following supporting information can be downloaded at: https://www.mdpi.com/article/10.3390/nu14214460/s1, Figure S1: Funnel plots representing publication bias in the studies reporting the effect of walnut consumption on (A) TC (mg/dL), (B) LDL−C (mg/dL), (C) HDL−C (mg/dL) and (D) TG (mg/dL).
