# Supplementary material for: The Effect of Walnut Intake on Lipids: A Systematic Review and Meta-Analysis of Randomized Controlled Trials
Source: Nutrients. 2022 Oct 23;14(21):4460. doi: 10.3390/nu14214460 (PMC9655834; doi:10.3390/nu14214460)
Supplement: Supplementary file 1 [file nutrients-14-04460-s001.zip › nutrients-1968873-supplementary.pdf]

Supplementary Materials: The Effect of Walnut Intake on Lipids: Systematic Review and Meta-Analysis of Randomized Controlled Trials

Saeed Mastour Alshahrani, Reham M Mashat, Diaa Almutairi, Alaa Mathkour, Sahar Saad Alqahtani, Amirah Alasmari, Abdullah Hassan Alzahrani, Reem Ayed, Mohammed Yahya Asiri, Alsanussi Elsherif and Abdullah Alsabaani

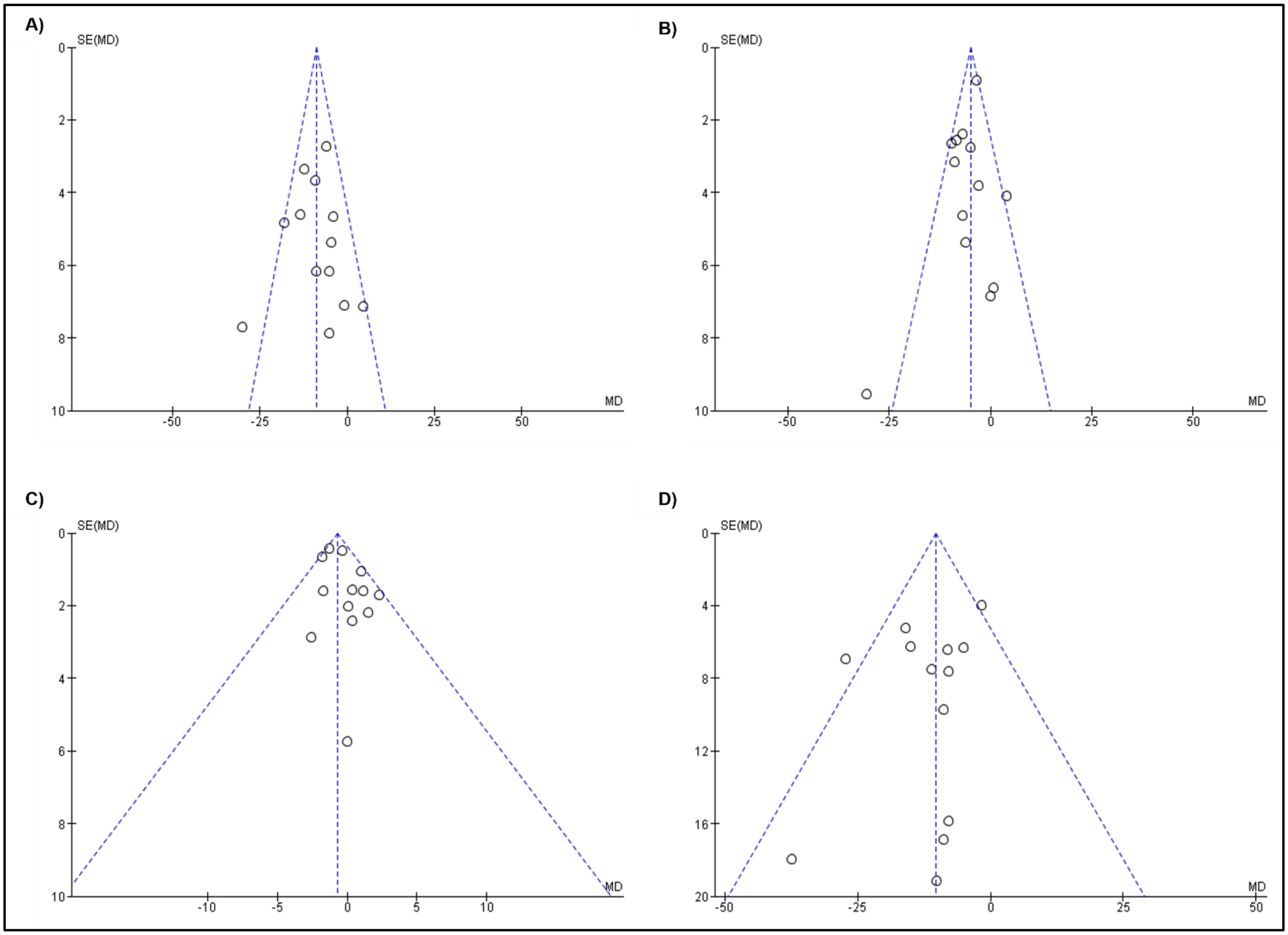

Figure S1. Funnel plots representing publication bias in the studies reporting the effect of walnut consumption on **(A)** TC (mg/dL), **(B)** LDL-C (mg/dL), **(C)** HDL-C (mg/dL) and **(D)** TG (mg/dL).
